# Fine-Tuning Directional Message Passing Neural Networks: Predicting Properties of Conjugated Organic Polymers with High Accuracy

**DOI:** 10.3390/polym18070879

**Published:** 2026-04-02

**Authors:** Igor P. Koskin, Lev S. Petrosyan, Maxim S. Kazantsev

**Affiliations:** 1 N.N. Vorozhtsov Novosibirsk Institute of Organic Chemistry, Siberian Branch of the Russian Academy of Sciences, 630090 Novosibirsk, Russia; osingran@yandex.ru (I.P.K.); l.petrosyan@g.nsu.ru (L.S.P.); 2 Faculty of Natural Sciences, Novosibirsk State University, 630090 Novosibirsk, Russia

**Keywords:** graph neural network, learning transfer, directional message passing, polymer informatics, electronic property prediction, fine-tuning

## Abstract

Conjugated organic polymers are the cornerstone of modern organic electronics, yet accurate prediction of their properties remains a challenging task due to their synthetic complexity and high computational cost of quantum-chemical methods. Here, we develop a graph neural network based on the DimeNet++ direct message passing architecture to predict HOMO, LUMO and energy gaps of conjugated polymers directly from their 3D monomer structure. The model was pre-trained on TD-DFT-extrapolated data and trained on a limited dataset of experimentally measured properties. As a result, pre-training had significantly improved model’s accuracy compared to direct training (MAEs ~0.3 eV vs. 0.074 eV, 0.141 and 0.172 for HOMO/LUMO and energy gap, respectively). Pre-training on monomer DFT data did not provide comparable gains. The results demonstrate that polymer-relevant pre-training is critical for capturing structure–property relationships and enables accurate predictions without delta-learning or prior quantum-chemical calculations, facilitating efficient screening and rational design of conjugated polymers for organic optoelectronics.

## 1. Introduction

Lightweight, flexible and low-cost conjugated organic polymers with potential applications ranging from organic solar cells [1,2,3], field-effect transistors [4,5,6] and photo/electroluminescent diodes [7] to sensors [8,9,10], organic batteries [11] and supercapacitors [12,13] are an undeniable cornerstone of modern organic optoelectronics. However, molecular design and screening for novel prospective polymeric materials offer a significant challenge due to synthetic and experimental difficulties associated with a trial-and-error approach. Exacerbating the issue, quantum-chemical modelling, usually used to assist in screening, is equally challenging for conjugated polymers with various methods either struggling to adequately capture and describe the inherently supramolecular nature of polymers without fine-tuning (DFT/TD-DFT) [14,15,16] or impractical on a large scale owing to their high computational costs (GW approximation) [17,18]. At the same time, various public libraries of conjugated organic polymers [19] containing thousands of structural and property datapoints—such as energy gaps, HOMO/LUMO energies, electrochemical data, glass transition temperatures, optical and mechanical functionalities as well as many others—are readily available. Wide availability of experimental information serves as a perfect platform for a data-driven approach and machine learning (ML), allowing researchers to accurately predict the desired property of target materials while circumventing common bottlenecks such as chemical synthesis and experimental measurements as well as quantum-chemical modelling.

In recent years, ML has been extensively used in the field of organic optoelectronics, allowing one to achieve significant progress in modelling and predict a wide array of organic material properties ranging from optoelectronic descriptors, such as energies of boundary orbitals and energy gap [20,21,22,23,24,25], UV-Vis spectra [26,27,28], and photoluminescence quantum yields [29,30,31,32], to physical ones such as phase transition temperature [33,34], Young’s modulus [35,36], and reaction products thermodynamical properties [37,38,39]. Over time, various ML approaches were used to predict properties of organic polymers with MAE values for energy gaps reported as random forest (0.12–0.43 eV) [40], support vector regression (0.18–0.35 eV) [41], and convolutional neural networks (0.14–0.40 eV), with an MAE lower than 0.1 eV still remaining an elusive target.

One of the most rapidly advancing approaches in ML are graph neural networks (GNNs) [42,43,44,45,46,47] designed to utilize graphs as the input values, thus allowing us to achieve success in the fields of identifying new drugs and biologically active molecules, modelling physical systems, and designing new molecules. Out of possible GNN architectures, Directional Message Passing Neural Networks (DMPNN)—such as the DimeNet++ model—are especially promising for computational chemistry applications as they are explicitly tailored to accurately predict quantum-chemical properties of molecules. Due to their inherent nature, molecular graphs enable the transfer of structural information to the neural network, thereby improving the quality and contextual relevance of the overall prediction. Various approaches of molecular graph vectorization allow the extraction of different properties (e.g., bond lengths, angles and dihedral angles between bonds), which are relevant to the task, and can lead to a different predictive quality of the overall model. Consequently, utilization of a full 3D molecular structure encoded as a molecular graph has previously shown to net an overall improvement in the prediction quality in the case of small molecules. In previous years, several works have shown the utilization of 3D molecular structures as the input data for ML models in the field of materials science, such as GNNs with special descriptors [48], atomistic line GNNs [49], and 3D convolutional neural networks [50]. However, they have yet to be utilized as the input values for an ML model in the case of organic polymers.

Some of the key parameters to ascertain the functionality of conjugated organic polymers as prospective materials for optoelectronic devices are the energy gap (Eg), defined as the difference between HOMO and LUMO, as well as HOMO/LUMO energies themselves. For instance, the energy gap directly influences absorption/emission bands [16] as well as photoluminescence quantum yield and quenching mechanisms [51], while HOMO/LUMO energies are important parameters for electron and hole injection [52], redox stability and overall chemical properties of organic polymers. Knowing the energy of boundary orbitals as well as the energy gap in advance is paramount for targeted molecular design of polymeric compounds with desired and advanced properties, e.g., conjugated polymers with the absorption/emission bands shifted towards red or infrared regions allowing higher efficiency of energy harvesting for solar cells. Due to the aforementioned difficulties associated with ab initio computational prediction of the energy gap and energy levels of polymers, machine learning is often the only available approach to evaluate these properties prior to time-consuming synthesis and experimental measurements.

ML models have been successfully utilized to readily predict optoelectronic and structural properties of conjugated organic polymers varying from boundary orbital energies and energy gap to glass transition temperatures and elasticity. A delta-learning approach is of particular interest since it allows one to achieve accuracy of prediction as low as 0.07 eV, as was demonstrated for HOMO/LUMO energies and energy gap for a wide database of conjugated organic polymers. However, one downside of such an approach is that in order to predict properties of a material, one must first estimate its properties by other means (such as molecular mechanics/dynamics or DFT calculations)—which is often a limiting step if property screening is necessary.

In this work, we have developed a novel GNN utilizing DMPNN DimeNet++ architecture and the 3D molecular structure of monomers derived from molecular mechanics as the input for the molecular graph, which allowed us to predict energy gap and HOMO and LUMO energy levels of the organic conjugated polymers with high accuracy. To achieve this, we pre-trained our GNN model on the dataset containing the energy gap and HOMO/LUMO energies of monomers with structural characteristics similar to the studied conjugated polymers. This pre-training allowed us to significantly increase accuracy when compared to a model trained solely on polymeric data, reaching a mean accuracy of 0.07 eV, which is on par with the state-of-the-art models in the field. Unlike previous examples, high predictive accuracy of the presented model was achieved without a delta-learning approach, thus bypassing time-consuming TD-DFT pre-calculation.

## 2. Materials and Methods

### 2.1. Dataset Preparation

**Figure 1:**
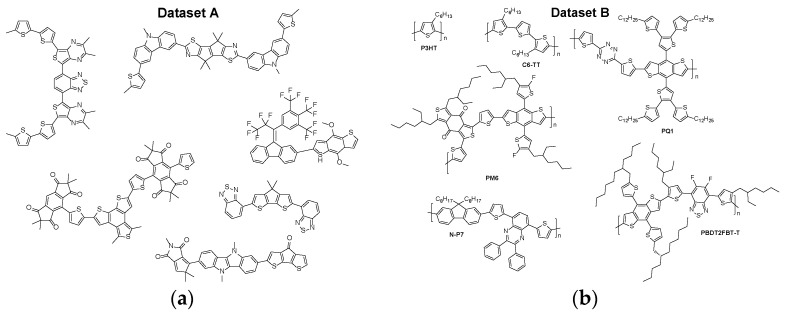
Representative examples of molecules and polymers found in (**a**) *Dataset A*; (**b**) *Dataset B*.

Two datasets were adopted for the purpose of model training: *Dataset A* [53] and *Dataset B* [19]. *Dataset A* contained ~60,000 datapoints: HOMO/LUMO and energy gap of monomers calculated at B3LYP/6-31G* level of theory (*Dataset A-DFT*) as well as HOMO/LUMO and energy gap energies extrapolated from a single monomer to a series of oligomers and then to a polymer limit utilizing the TD-DFT/B3LYP/6-31G* level of theory (*Dataset A-TD*). *Dataset B* contained ~1300 datapoints of experimentally measured HOMO/LUMO and energy gap of conjugated organic polymers. *Dataset A-TD* was truncated to ~54,000 datapoints, cutting out molecules with energy gaps lower than 1.0 eV and higher than 2.4 eV to ensure better overlap between *Dataset A-TD* and *Dataset B* (see Table S2 in Supplementary Materials for additional information). Additionally, duplicate datapoints were removed from *Dataset B*. Box-Cox transformation was applied to every dataset in order to convert it from non-normal form into a normal distribution, which stabilizes variance and improves fitting for regression models. Each dataset was split into training, testing and validation subsets at an 8:1:1 ratio. A representative example of typical compounds found in *Dataset A* and *Dataset B*, respectively, can be seen in Figure 1.

### 2.2. GNN Methods

All machine learning models presented in this work utilize Directional Message Passing Neural Networks (DMPNN) with DimeNet++ architecture—a graph neural network specifically tailored to accurately predict physical and quantum properties of molecules. Unlike other graph neural networks, DimeNet++ utilizes geometrical information explicitly, which allows the model to fully take molecular geometry (bond lengths, angles and dihedral angles) into account. DimeNet++ is invariant to transformations (such as translations and rotations) which is crucial for the correct processing of molecular structures. Finally, DimeNet++ features directional message passing which improves the model’s ability to take into account the local chemical surroundings of each atom. In summary, DimeNet++ is a well-optimized and fast architecture that combines high predictive accuracy and relatively low training overhead.

**Figure 2:**
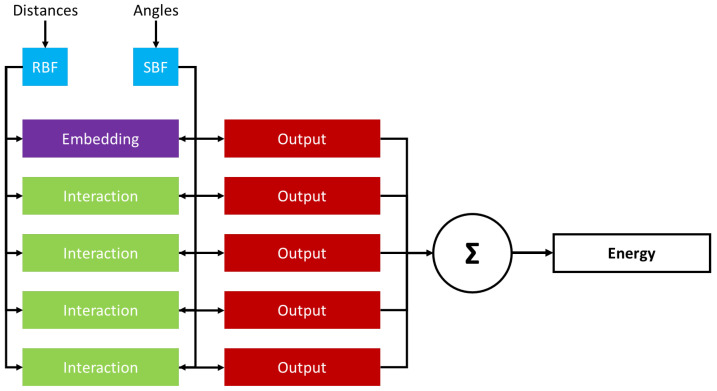
Schematic graphical representation of DimeNet++ architecture. RBF and SBF stand for radial basis functions and spherical basis functions, respectively. Violet, green and red rectangles denote embedding, interaction and output layers of the architecture, black arrows depict information and data passing between the layers. Σ symbol represents summation of output values across all the output layers.

DimeNet++ architecture (Figure 2) utilized in this work consists of several key blocks and components. The first component of the architecture is the Embedding Block that constructs initial features (embedding) based on the input atomic types, coordinates and connectivity. The second component is the Interaction Blocks that perform message passing, aggregation, and iteratively refine embeddings by utilizing information from neighbouring atoms and distances. In order to embed angular data, the DimeNet++ architecture employs a two-hop geometric message passing approach that handles interactions between an edge and its neighbouring edges. The last block is the Output Block that converges and processes refined embeddings in order to predict a desired molecular property.

Atomic numbers (as node features) and atomic coordinates were used as the input data for all of the featured models. Input molecular geometries for compounds from *Dataset A* (TD-DFT/B3LYP/6-31G*) were used as is. In the case of *Dataset B*, molecular geometries of monomers were optimized by a MMFF94s molecular mechanics force field. The output data of the models is the predicted value of the target property (HOMO, LUMO, or energy gap) for each molecule. The resulting machine learning methods were validated through k-fold cross-validation, which is a technique necessary to evaluate the model’s generalization and ensure it is not overfitted. Optimized hyperparameters utilized for all presented DimeNet++ ML models can be found in Table S1 of Supplementary Materials.

## 3. Results

Since the explicit representation of a molecular geometry is crucial to capture and recreate the structure–property relation of organic conjugated polymers, we utilized the DMPNN approach, which represents 3D molecular geometry via a molecular graph form and thus allows the model to extract relevant structural data (such as connectivity, bond lengths, angles, and dihedral angles) out of it. DimeNet++ GNN architecture was previously shown to be effective at predicting HOMO/LUMO and the energy gap of small conjugated molecules as well as being a great compromise between the computational cost of training and the model’s resulting predictive ability. Therefore, we chose DimeNet++ as the base model in our work.

**Figure 3:**
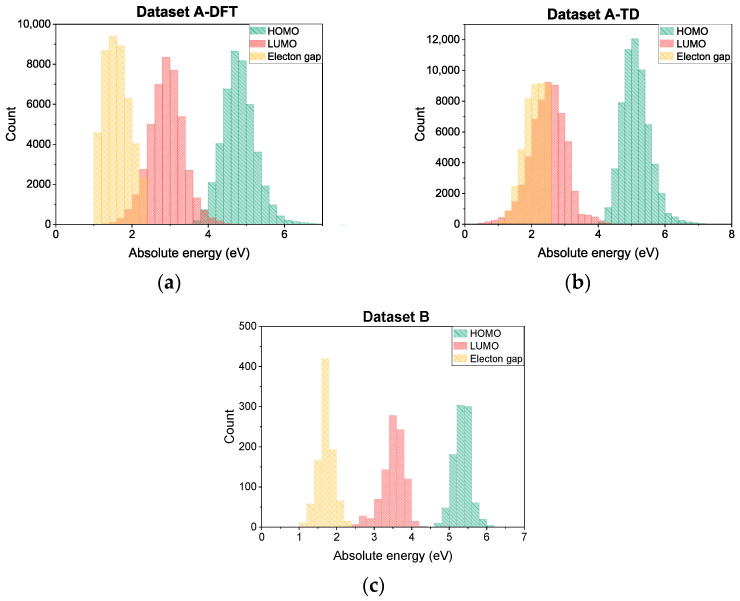
Datapoint value distributions depicted as column charts for: (**a**) *Dataset A-DFT*; (**b**) *Dataset A-TD*; (**c**) *Dataset B*. Green columns represent HOMO values, red columns—LUMO values, yellow—electron gap values.

For the process of model training, we adopted two different datasets: (1) *Dataset A* (~54,000 datapoints), containing molecular geometries, HOMO/LUMO and energy gap calculated at the DFT level of theory for single monomers (referred as *Dataset A-DFT*), as well as HOMO/LUMO and energy gap calculated at the TD-DFT level of theory by extrapolating a series of finite-length oligomers to an infinite polymer chain (referred as *Dataset A-TD*); (2) *Dataset B* (~1300 datapoints), containing experimentally measured HOMO/LUMO and energy gap for conjugated organic polymers. The value distribution for adopted datasets is shown in Figure 3.

**Figure 4:**
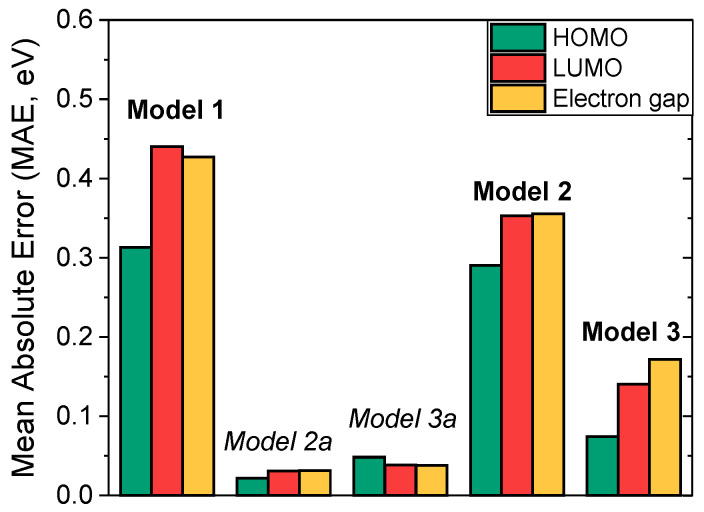
Mean absolute errors (MAE) on validation sets of HOMO (green column), LUMO (red column) and energy gap (yellow) energies for Model 1, Model 2a, Model 3a, Model 2 and Model 3—first, second, third, fourth and fifth sets of columns, respectively.

At the first step, the basic DMPNN **Model 1** was trained on the experimentally measured HOMO/LUMO and energy gaps for conjugated organic polymers from *Dataset B* (see Figure S1 in Supplementary Materials for an expected/predicted value distribution for the model). Accordingly, Model 1 exhibited MAEs of 0.3130 eV, 0.4401 eV and 0.4271 eV for HOMO, LUMO and energy gap, respectively (first set of columns in Figure 4).

**Model 2a** and **Model 3a** were pre-trained on the DFT data calculated for monomers and TD-DFT data extrapolated to the polymer limit (*Dataset A-DFT* and *Dataset A-TD-DFT*), respectively. As a result, **Model 2a** and **Model 3a** exhibited roughly similar MAEs (second and third set of columns on Figure 4) for all studied properties (Model 2a: 0.0220 eV, 0.0310 eV, 0.0315 eV; Model 3a: 0.0483 eV, 0.0383 eV, 0.0380 eV—for HOMO, LUMO and energy gap, respectively).

**Figure 5:**
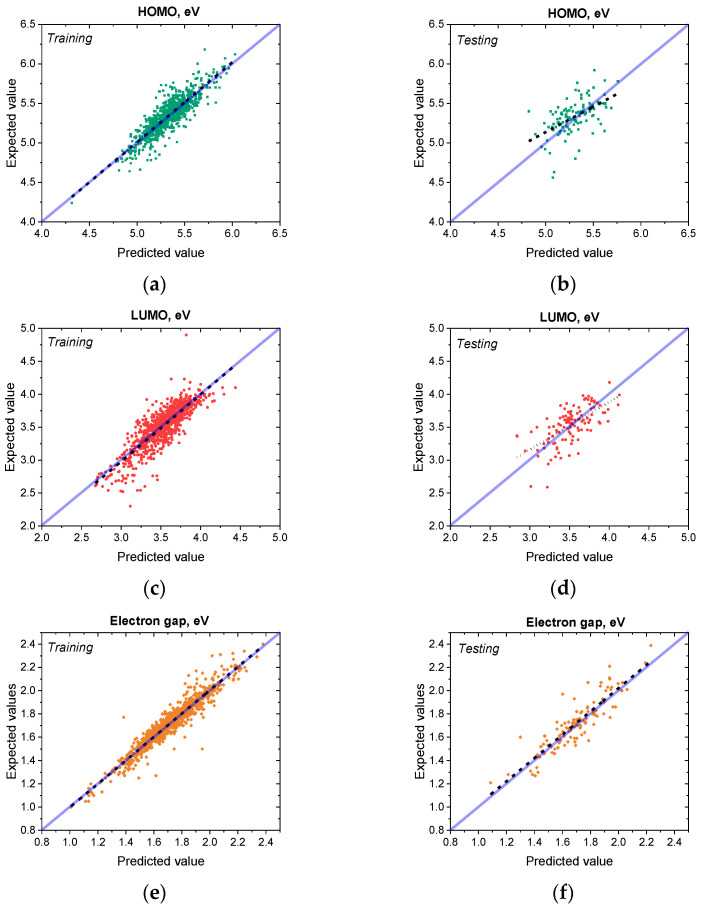
Overall performance and data distribution of Model 3a (X-axis—values, predicted by the model, Y-axis—expected values) for (**a**) HOMO energy on training dataset; (**b**) HOMO energy on testing dataset, (**c**) LUMO on training dataset; (**d**) LUMO on testing dataset; (**e**) energy gap on training dataset; (**f**) energy gap on testing dataset. Green, red and yellow dots depict datapoints for HOMO, LUMO and energy gap, respectively; dashed black line—linear approximation, solid semitransparent blue line—predicted value coincides with expected value (X = Y).

Finally, **Model 2** and **Model 3** were fine-tuned on the experimental data from *Dataset B*. Since *Dataset A* features molecular geometries optimized at the DFT level while the geometries from *Dataset B* were optimized with the molecular mechanics approach, we did not freeze any weights in the process of fine-tuning. In such a way, we allow both models to have enough internal flexibility to compensate for the inherent difference in the way molecular geometries were obtained. The resulting fine-tuned **Model 2** and **Model 3** demonstrated varying accuracy. **Model 2** exhibited MAEs of 0.2901 eV, 0.3531 eV and 0.3554 eV for HOMO, LUMO and energy gap, respectively, which signifies not only distinct lack of prediction accuracy but also clearly shows that the model failed to improve over the baseline through the course of fine-tuning. At the same time, **Model 3** (Figure 5), which was pre-trained on TD-DFT extrapolated data, showed vastly better MAEs of 0.0742 eV, 0.1407 eV and 0.1718 eV for energy gap, HOMO and LUMO, respectively. Additional statistical characterization of **Model 1** and **Model 3** as a way to demonstrate the increase in predictive quality of **Model 3** due to the pre-training process is presented in Table S3 of the Supplementary Materials.

## 4. Discussion

The achieved margin of error for **Model 1** (trained exclusively on experimental polymer data) is unsatisfactory and inapplicable for any valid estimation of conjugated organic polymers’ properties. This result can be attributed to the limited size of *Dataset B*, which makes it insufficient for a complex DimeNet++ architecture. Another factor potentially limiting the accuracy of **Model 1** is a high degree of measurement error commonly arising from the fact that different datapoints can be measured by different experimental approaches (e.g., absorption and/or luminescence spectroscopy, XPS, cyclic voltammetry). These limitations are hard to overcome as acquiring new datapoints to widen the dataset in any significant way requires time-consuming synthetic procedures and measurements. Therefore, we employed a different strategy to improve the predictive accuracy of further GNN models for conjugated organic polymers.

An alternative strategy to training the GNN model directly on experimentally measured HOMO/LUMO and energy gaps of conjugated organic polymers is the fine-tuning approach, which constitutes pre-training on a wider dataset and then the final training on a narrower dataset with or without freezing pre-trained model weights. This approach not only allowed us to overcome the lack of datapoints in the dataset but also improved the overall accuracy of the model. As stated before, both **Model 2** and **Model 3** exhibit significant improvement over **Model 1** in terms of predictive quality, which demonstrates that both of the models’ architectures were adequate for the models to capture and approximate the underlying connection between monomer’s geometry and boundary orbital energies.

An observed difference in predictive quality between **Model 2** and **Model 3** clearly shows that pre-training on TD-DFT extrapolated data—unlike DFT calculated data for single monomers—allows the model to more accurately capture the polymeric nature of studied materials and thus improve overall predictive quality. Therefore, we can conclude that pre-training the model on TD-DFT extrapolated data and then fine-tuning it without freezing any of the internal layers is the most efficient strategy to achieve a high predictive accuracy.

Further analysis (Figures S1–S3 of Supplementary Materials) of **Model 3a** and **Model 3** through the UMAP procedure (Uniform Manifold Approximation and Projection), which projects a complex multidimensional space of a ML model into a 2D graph depicting an internal neighbouring structure with regards to the atom type and its contribution to the resulting energy, allowed us to show that fine-tuning process have not changed the distribution significantly, which in turn is a sign of the model adapting the data from pre-training into a new domain of information instead of changing drastically. The fact that observed differences are more noticeable in the Euclidean metric than in the Cosine metric (given the general similarity of the cosine UMAPs) means that fine-tuning has changed the scales/norms of embeddings and local distances more than the direction of representations, which is a sign of soft adaptation: the semantics of the latent model are generally preserved, but some groups of points are compacted and shifted. Observed (via UMAP analysis) uninterrupted learning transfer between **Model 3a** and **Model 3** may also suggest some level of generalization achieved on the studied subsection of the chemical space of linear, conjugated organic polymers; however, an in-depth investigation beyond the scope of this work is necessary to access it.

As in its final state, **Model 3** does not explicitly take into account any potential supramolecular polymeric effects such as regioregularity, tacticity, or side-chain emergence, as the only information fed into the input layer of the architecture is the 3D geometry of a monomer. As a result of the training and validation process, the model was still indirectly influenced by these effects due to fitting output results on the dataset of experimentally measured data. However, a more direct inclusion of supramolecular parameters as input data may act as a potential way to improve predictive accuracy of ML models for organic conjugated polymers. Other ways to increase the model’s predictive quality and performance include increasing the amount of datapoints in the experimental dataset, which can improve the model’s generalization across the conjugated organic polymers chemical space, as well as substituting the MMFF94s method to optimized monomer 3D geometries for a more sophisticated and accurate DFT-based approach to achieve better correspondence between the model’s pre-training and training. Both of the aforementioned factors remain as key bottlenecks for the presented model’s performance.

The resulting accuracy of **Model 3** (~0.07 eV for energy gap) is comparable with the state-of-the-art results achieved using NN for conjugated organic polymers, such as those reported in the (~0.06 eV for energy gap) [54]. While the resulting high accuracy of Model 3 is not strictly meaningful in terms of its predictive quality, as it was trained on the dataset that features experimentally measured datapoints with different and relatively high margins of error, it is still indicative of the model’s overall performance and, hence, applicability of presented architecture to other classes and groups of polymers if provided pre-training and training datasets of acceptable quality. Unlike the previously utilized delta-learning approach, which entails calculating the energy gap and HOMO/LUMO at the TD-DFT level of theory as the first step and then estimating the value of error through a trained machine learning model, our model obtained via fine-tuning strategy can predict energy gap and HOMO/LUMO energies directly from a chemical structure of a monomer without any necessity to optimize it first. Therefore, bypassing a time-consuming TD-DFT calculation step vastly improves applicability of our model to fast and accurate screening to facilitate targeted molecular design of prospective conjugated organic polymers for organic optoelectronics, photovoltaics and sensorics.

## 5. Conclusions

In summary, we demonstrated a ML model based on DMPNN DimeNet++ architecture capable of predicting HOMO/LUMO and the energy gap of conjugated polymers directly from their 3D monomer structure with a high degree of accuracy. While direct training of DimeNet++ model on a limited experimental dataset resulted in unsatisfactory predictive performance (MAEs > 0.3 eV), the pre-training approach was shown to significantly improve the model’s quality. Pre-training on TD-DFT extrapolated data followed by training on an experimental dataset was shown to be the most efficient approach resulting in mean absolute errors as low as 0.074 eV for energy gap, 0.141 eV for HOMO, and 0.172 eV for LUMO, respectively. Importantly, unlike delta-learning approaches, the proposed model does not require prior quantum-chemical calculations for each new structure, thereby eliminating the need for time-consuming TD-DFT pre-computations. Consequently, the results confirm that incorporating polymer-relevant pre-training data is critical for capturing the intrinsic structure–property relationships of conjugated polymers. Therefore, this work establishes an efficient framework for conjugated polymer screening and targeted molecular design though the utilization of pre-trained graph neural network architecture to overcome data scarcity and potential computational bottlenecks.

## Data Availability

All ML models’ weights presented in this work; the raw data regarding ML training can be found in an open GitHub repository: https://github.com/Levitsiy/PolymersPropertiesPrediction, accessed on 30 March 2026.

## Supplementary Materials

The following supporting information can be downloaded at: https://www.mdpi.com/article/10.3390/polym18070879/s1, Table S1. Optimized hyperparameters utilized for all the presented DimeNet++ ML models in this work; Table S2. Characteristics of datasets utilized in this work (points—the amount of datapoints in a dataset, min/max–lowest and highest value of said property in a dataset); Table S3. Statistical characterization of Models 1 and 3 cross-validation for HOMO, LUMO and Eg (energy gap) properties. The cross validation was performed over 10 different folds, the resulting statistical characteristics were averaged out (mean column), standard deviation (std dev) and confidence intervals (ci_low and ci_high) were also calculated. Statistical characteristics include: MAE (mean absolute error), RMSE (root mean square error) and R2 (coefficient of determination); Figure S1. UMAP projections of **Model 3a** (pre-training) and **Model 3** (final model) multidimensional internal spaces into 2D Euclidian and cosine spaces in the case of HOMO energy prediction: top-right corner—cosine space projection for **Model 3a**, top-left corner—cosine space projection for **Model 3**, bottom-right corner—**Model 3a** Euclidian space projection, bottom-left corner—**Model 3** Euclidian space projection; Figure S2. UMAP projections of **Model 3a** (pre-training) and **Model 3** (final model) multidimensional internal spaces into 2D Euclidian and cosine spaces in the case of LUMO energy prediction: top-right corner—cosine space projection for **Model 3a**, top-left corner—cosine space projection for **Model 3**, bottom-right corner—**Model 3a** Euclidian space projection, bottom-left corner—**Model 3** Euclidian space projection; Figure S3. UMAP projections of **Model 3a** (pre-training) and **Model 3** (final model) multidimensional internal spaces into 2D Euclidian and cosine spaces in the case of band gap energy prediction: top-right corner—cosine space projection for **Model 3a**, top-left corner—cosine space projection for **Model 3**, bottom-right corner—**Model 3a** Euclidian space projection, bottom-left corner—**Model 3** Euclidian space projection.

## Abbreviations

The following abbreviations are used in this manuscript:

ML	Machine Learning
GNN	Graph Neural Network
DMPNN	Direct Message Passing Neural Network
DFT	Density Functional Theory
TD-DFT	Time-Dependent Density Functional Theory
HOMO	Highest Occupied Molecular Orbital
LUMO	Lowest Occupied Molecular Orbital
MAE	Mean Absolute Error
UMAP	Uniform Manifold Approximation and Projection
