# Supplementary material for: Fine-Tuning Directional Message Passing Neural Networks: Predicting Properties of Conjugated Organic Polymers with High Accuracy
Source: Polymers (Basel). 2026 Apr 2;18(7):879. doi: 10.3390/polym18070879 (PMC13075011; doi:10.3390/polym18070879)
Supplement: Supplementary file 1 [file polymers-18-00879-s001.zip › polymers-4206825-supplementary.pdf]

Supplementary Information

Fine-tuning directional message passing neural networks: predicting properties of conjugated organic polymers with high accuracy

Igor P. Koskin, Lev S. Petrosyan and Maxim S. Kazantsev

Table S1. Optimized hyperparameters utilized for all the presented DimeNet++ ML models in this work.

Hyperparameter	Description	Value
hidden_channels	Hidden representation (embedding) dimension	128
out_channels	Output layer dimension	1
num_blocks	Number of interaction blocks	4
int_emb_size	Dimension of intermediate embeddings in interaction blocks	64
basis_emb_size	Dimension of basis function embeddings	16
output_blocks	Number of output blocks	3
num_spherical	Number of spherical harmonics (angular component)	3
num_radial	Number of radial basis functions	2
cutoff	Cutoff radius for interatomic interactions	5
learning_rate	Learning speed	0.008
batch_size	Mini-batch size	64

Table S2. Characteristics of datasets utilized in this work (points – the amount of datapoints in a dataset, min/max – lowest and highest value of said property in a dataset).

		Points	Min	Max	Source
**Dataset A-DFT**	HOMO	54250	3.26	7.58	https://data.nlr.gov/submissions/236
	LUMO		0.49	5.05	
	Electron gap		1.00	2.40	
**Dataset A-TD**	HOMO	54250	3.87	8.07	https://data.nlr.gov/submissions/236
	LUMO		0.65	4.68	
	Electron gap		1.00	2.40	
**Dataset B**	HOMO	1324	4.24	6.18	10.1021/acs.jpclett.8b00635
	LUMO		2.30	4.90	
	Electron gap		1.00	2.40	

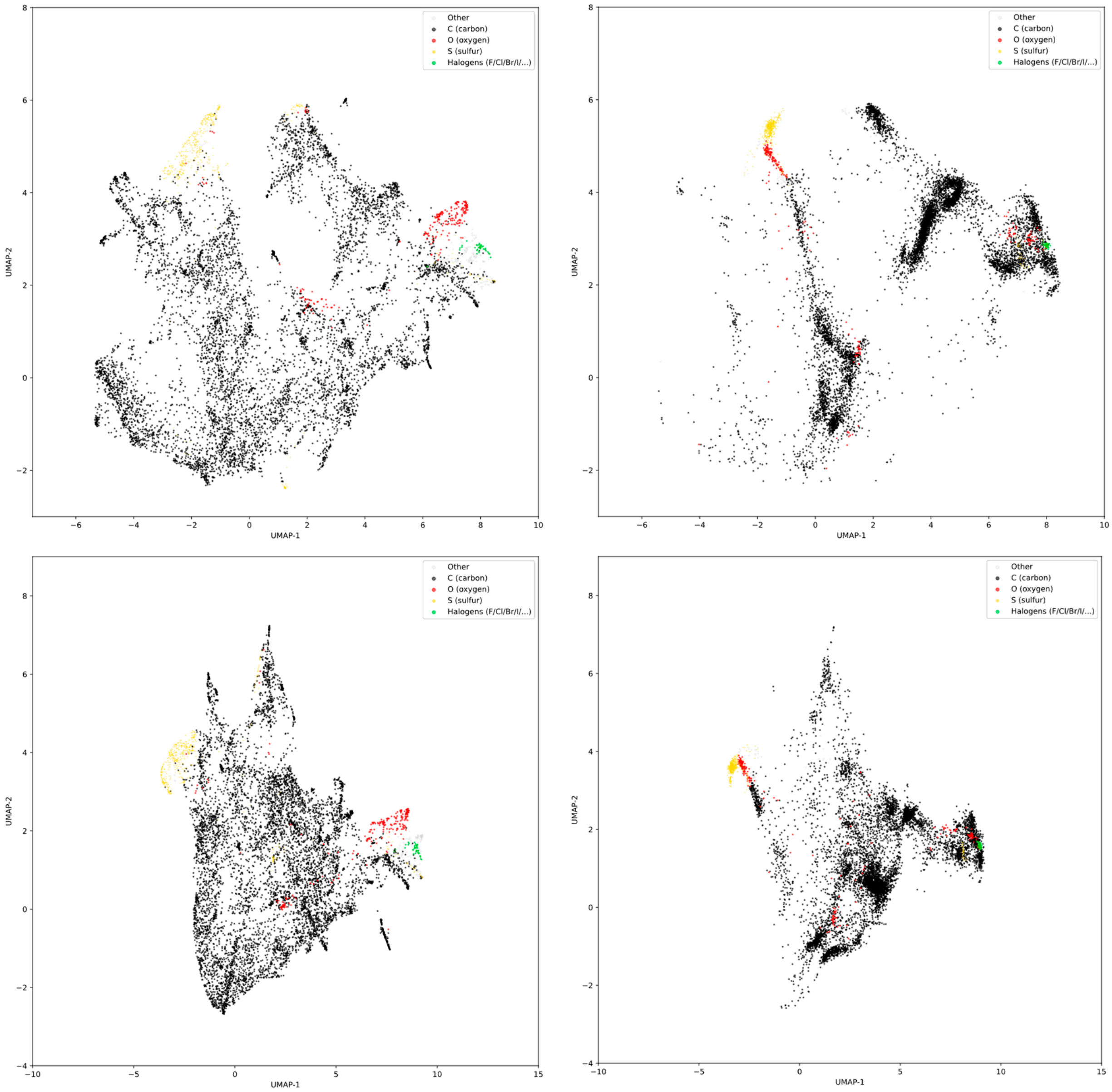

Figure S1. UMAP projections of **Model 3a** (pre-training) and **Model 3** (final model) multidimensional internal spaces into 2D Euclidian and cosine spaces in the case of HOMO energy prediction: top-right corner – cosine space projection for **Model 3a**, top-left corner – cosine space projection for **Model 3**, bottom-right corner – **Model 3a** Euclidian space projection, bottom-left corner – **Model 3** Euclidian space projection.

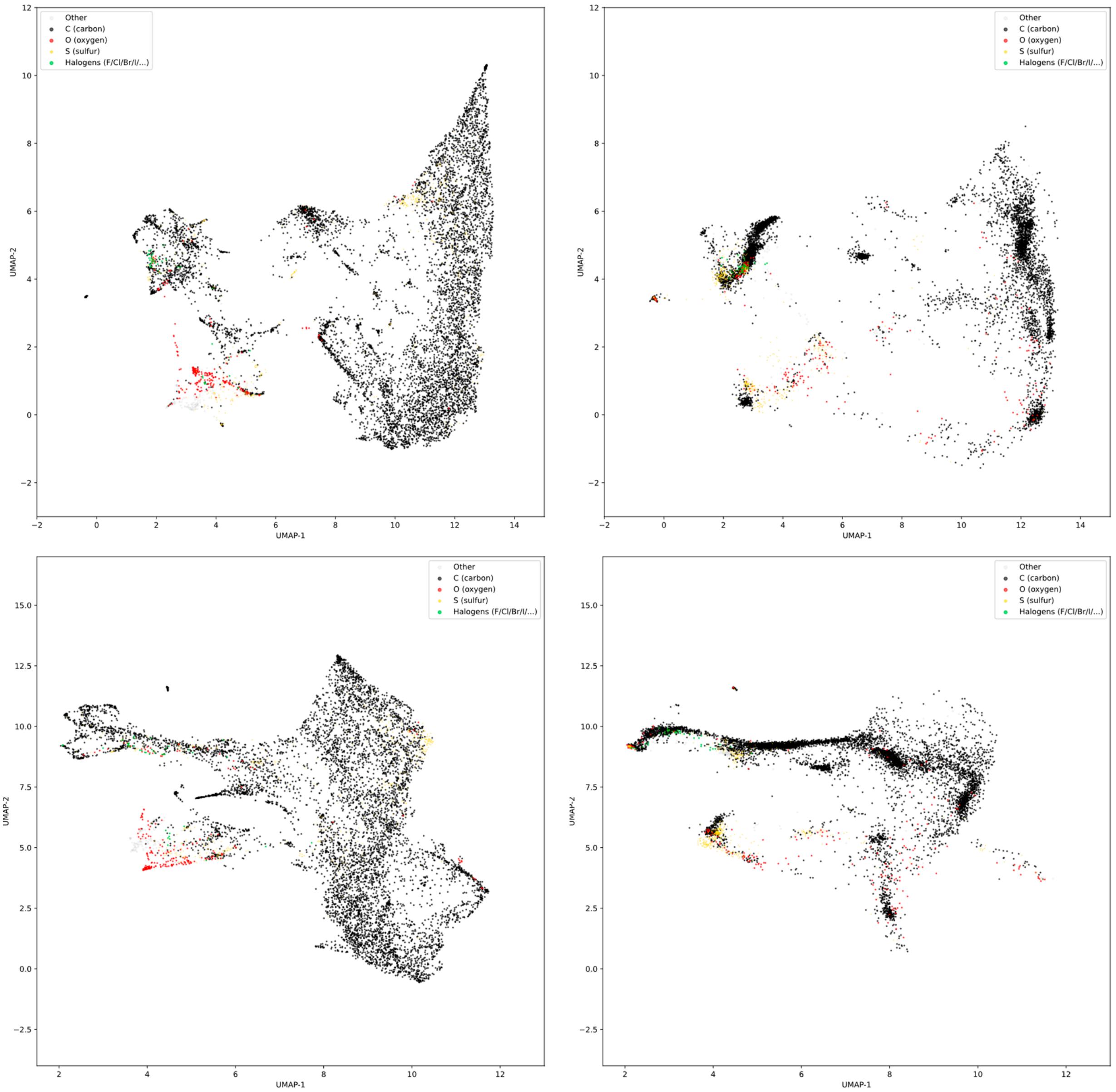

Figure S2. UMAP projections of **Model 3a** (pre-training) and **Model 3** (final model) multidimensional internal spaces into 2D Euclidian and cosine spaces in the case of LUMO energy prediction: top-right corner – cosine space projection for **Model 3a**, top-left corner – cosine space projection for **Model 3**, bottom-right corner – **Model 3a** Euclidian space projection, bottom-left corner – **Model 3** Euclidian space projection.

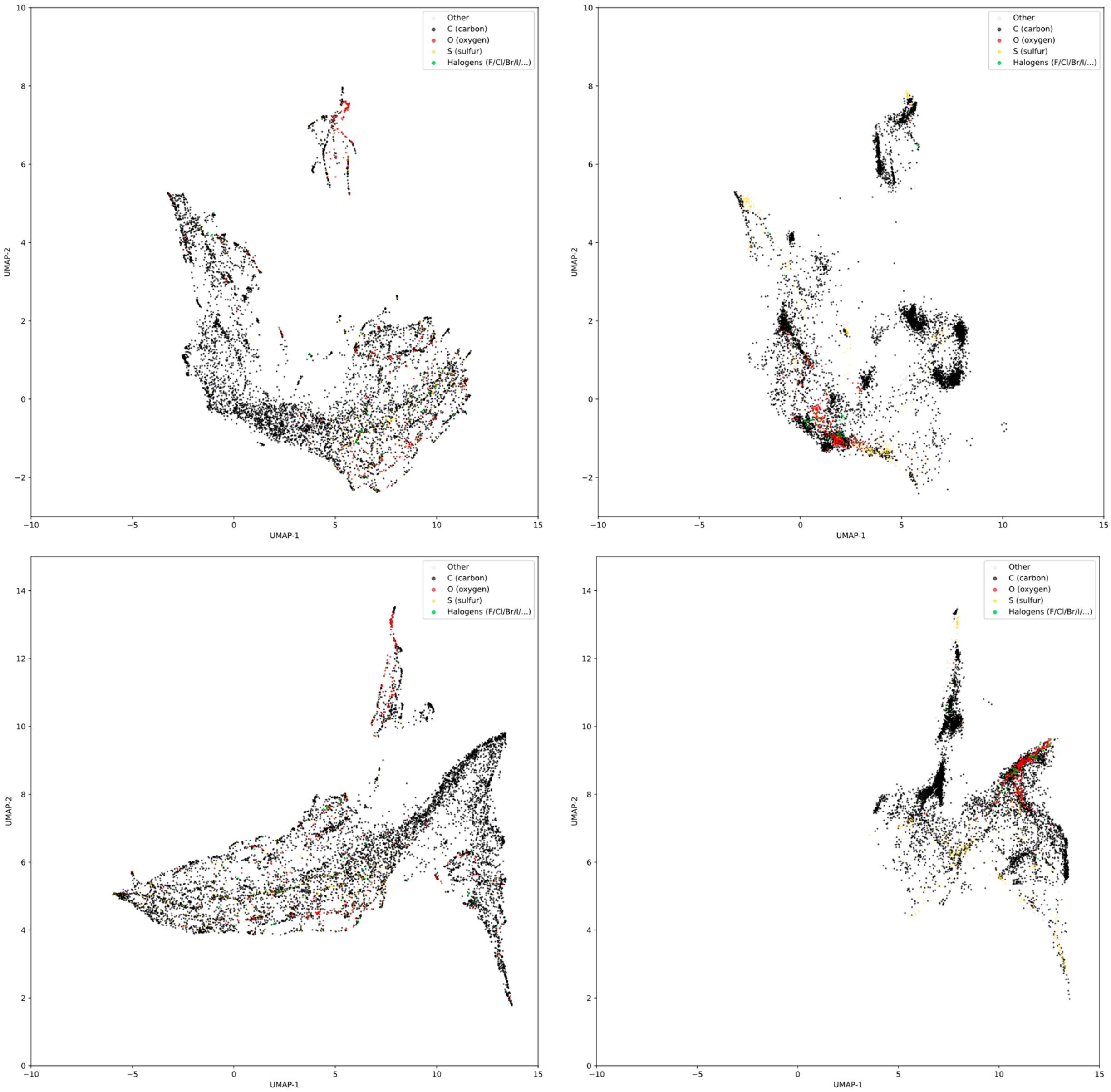

Figure S3. UMAP projections of **Model 3a** (pre-training) and **Model 3** (final model) multidimensional internal spaces into 2D Euclidian and cosine spaces in the case of band gap energy prediction: top-right corner – cosine space projection for **Model 3a**, top-left corner – cosine space projection for **Model 3**, bottom-right corner – **Model 3a** Euclidian space projection, bottom-left corner – **Model 3** Euclidian space projection.

Table S3. Statistical characterization of Models 1 and 3 cross-validation for HOMO, LUMO and Eg (energy gap) properties. The cross validation was performed over 10 different folds, the resulting statistical characteristics were averaged out (mean column), standard deviation (std dev) and confidence intervals (ci_low and ci_high) were also calculated. Statistical characteristics include: MAE (mean absolute error), RMSE (root mean square error) and R^2 (coefficient of determination).

	**MODEL 1**																							
	MAE								RMSE								R^2							
	Training				Validation				Training				Validation				Training				Validation			
	mean	std dev	ci_low	ci_high	mean	std dev	ci_low	ci_high	mean	std dev	ci_low	ci_high	mean	std dev	ci_low	ci_high	mean	std dev	ci_low	ci_high	mean	std dev	ci_low	ci_high
HOMO	0.1499	0.0036	0.1473	0.1525	0.1550	0.0121	0.1464	0.1637	0.1988	0.0029	0.1967	0.2009	0.2033	0.0154	0.1923	0.2142	0.1920	0.0204	0.1774	0.2066	0.1416	0.0578	0.1002	0.1829
LUMO	0.1743	0.0052	0.1706	0.1780	0.1890	0.0204	0.1744	0.2036	0.2372	0.0080	0.2315	0.2429	0.2576	0.0318	0.2349	0.2803	0.3930	0.0361	0.3671	0.4188	0.2610	0.1303	0.1678	0.3542
Eg	0.1067	0.0073	0.1015	0.1119	0.1171	0.0124	0.1082	0.1259	0.1442	0.0081	0.1384	0.1500	0.1587	0.0190	0.1452	0.1723	0.5069	0.0542	0.4682	0.5457	0.3992	0.0990	0.3284	0.4700
	**MODEL 3**																							
	MAE								RMSE								R^2							
	Training				Validation				Training				Validation				Training				Validation			
	mean	std dev	ci_low	ci_high	mean	std dev	ci_low	ci_high	mean	std dev	ci_low	ci_high	mean	std dev	ci_low	ci_high	mean	std dev	ci_low	ci_high	mean	std dev	ci_low	ci_high
HOMO	0.0881	0.0104	0.0806	0.0955	0.1409	0.0114	0.1328	0.1491	0.1199	0.0129	0.1107	0.1291	0.1944	0.0173	0.1821	0.2068	0.7035	0.0638	0.6578	0.7491	0.2024	0.1617	0.0868	0.3181
LUMO	0.1220	0.0177	0.1001	0.1440	0.1913	0.0179	0.1691	0.2136	0.1667	0.0241	0.1368	0.1966	0.2571	0.0381	0.2099	0.3044	0.7002	0.0780	0.6033	0.7970	0.2769	0.0994	0.1535	0.4003
Eg	0.0369	0.0064	0.0323	0.0415	0.0842	0.0080	0.0785	0.0900	0.0510	0.0089	0.0446	0.0574	0.1213	0.0144	0.1110	0.1316	0.9370	0.0221	0.9212	0.9528	0.6462	0.0699	0.5963	0.6962
